# Exploring the related factors of fertilization failure in in-vitro fertilization-embryo transfer

**DOI:** 10.12669/pjms.42.4.13188

**Published:** 2026-04

**Authors:** Jiajia Liu, Yan Qin, Qi Song, Chen Li, Rongrong Liu, Hongzhi Shi

**Affiliations:** 1 Jiajia Liu, Department of Reproductive Medicine, Maternity & Child Care Center of Qinhuangdao, Qinhuangdao 066000, Hebei, China; 2 Yan Qin, Department of Reproductive Medicine, Maternity & Child Care Center of Qinhuangdao, Qinhuangdao 066000, Hebei, China; 3 Qi Song, Department of Reproductive Medicine, Maternity & Child Care Center of Qinhuangdao, Qinhuangdao 066000, Hebei, China; 4 Chen Li, Department of Reproductive Medicine, Maternity & Child Care Center of Qinhuangdao, Qinhuangdao 066000, Hebei, China; 5 Rongrong Liu, Children Protect Branch, Maternity & Child Care Center of Qinhuangdao, Qinhuangdao 066000, Hebei, China; 6 Hongzhi Shi, Department of Reproductive Medicine, Maternity & Child Care Center of Qinhuangdao, Qinhuangdao 066000, Hebei, China

**Keywords:** Fertilization failure, In Vitro Fertilization-Embryo transfer, Predictive factors

## Abstract

**Objective:**

To investigate the influencing factors of fertilization failure in in vitro fertilization-embryo transfer (IVF-ET), so as to conduct clinical predictions and reduce the fertilization failure rate.

**Methodology:**

A retrospective study was conducted on 1,818 IVF-ET cycles with natural sperm-egg fusion performed in the Department of Reproductive Medicine at the Maternity & Child Care Center of Qinhuangdao from January 2022 to December 2024. These cycles were divided into two groups: the experimental group (218 cycles) with fertilization failure-related adverse outcomes and the control group (1,600 cycles) with normal fertilization. Univariate analysis was first applied to compare general characteristics, endocrine parameters, gonadotropin-related indices, oocyte and sperm parameters, and infertility causes between the two groups. Subsequently, factors with a statistical difference (P < 0.05) in the univariate analysis were enrolled in a multivariate logistic regression model to identify independent influencing factors of fertilization failure.

**Results:**

Univariate analysis showed that infertility duration, primary infertility, non-tubal infertility factors, serum estradiol (E2) and luteinizing hormone (LH) levels on the day of human chorionic gonadotropin (HCG) administration, total oocyte yield, sperm motility, normal sperm morphology rate, and post-processing sperm concentration were all associated with IVF fertilization failure. Multivariate logistic regression analysis further identified primary infertility, non-tubal infertility factors, and elevated LH levels on the day of HCG administration as independent risk factors for fertilization failure.

**Conclusion:**

Clinical attention should be prioritized for female patients with primary infertility caused by non-tubal factors and elevated LH levels on the day of HCG administration, as these factors independently increase the risk of fertilization failure; targeted clinical interventions for such cases may help reduce the incidence of fertilization failure in IVF-ET cycles.

## INTRODUCTION

With the rapid development of assisted reproductive techniques, IVF-ET has become a beacon of hope for many infertile couples. However, the issue of fertilization failure continues to plague this field, which not only reduces the success rate of treatments but also imposes both physical and psychological burdens on patients. It is widely acknowledged that older patients and individuals with long-standing infertility often present with multiple reproductive system abnormalities. Prolonged infertility may trigger endocrine disorders and impair endometrial receptivity; additionally, psychological stress can exert negative effects on the reproductive endocrine system by disrupting the normal regulation of the hypothalamic-pituitary-gonadal axis, thereby exerting adverse impacts on fertilization. Yet our study demonstrated that these commonly concerned factors have no actual effect on fertilization outcomes, with female age, body mass index (BMI), male age, infertility duration, basal follicle-stimulating hormone (FSH), and anti-Müllerian hormone (AMH) all lacking predictive value for fertilization failure, a finding consistent with the results of several previous studies.^1^,^2^ Given the ambiguity surrounding the core influencing factors of fertilization failure in clinical practice, it is of significant clinical importance and urgent necessity to explore accurate predictive indicators for fertilization failure and clarify its independent risk factors, so as to provide a theoretical basis for clinical intervention and reduce the incidence of fertilization failure in IVF-ET cycles.^3^

## METHODOLOGY

This was a retrospective study. In this study, data from patients who underwent IVF-ET treatments with fertilization via natural sperm-egg fusion in the Department of Reproductive Medicine at Maternity & Child care center of Qinhuangdao from January 2022 to December 2024 were selected. Data were retrieved from the hospital information and management system, and all clinical and demographic information of the patients was collected.

### Inclusion criteria:

- Infertile couples receiving the first cycle of conventional IVF-ET with natural sperm-egg fusion in the study center during January 2022 to December 2024.
- Female patients with normal ovarian reserve function (basal FSH < 15 IU/L, AMH > 1.1 ng/mL) and regular menstrual cycles (21–35 days).
- Male patients providing fresh semen samples for insemination, without complete asthenospermia, necrospermia or azoospermia.
- Complete clinical and follow-up data available for the patients.

### Exclusion criteria:

- Patients undergoing intracytoplasmic sperm injection (ICSI), half-ICSI or other assisted fertilization methods.
- Patients complicated with severe systemic diseases (e.g., severe cardiovascular and cerebrovascular diseases, hepatic and renal insufficiency, autoimmune diseases, malignant tumors).
- Patients with mental disorders who were unable to cooperate with clinical treatment and follow-up.
- Patients with ovarian hyperstimulation syndrome (OHSS) during the treatment cycle.
- Treatment cycles with canceled oocyte retrieval or fertilization due to personal reasons or accidental factors.

### Ethical approval:

The study was approved by the Institutional Ethics Committee of Maternity & Child care center of Qinhuangdao (No.: QHDFY-2023031008; Date: March 10, 2023). Written informed consent was obtained from all participants prior to their enrollment in the study, and all research procedures were conducted in accordance with the Declaration of Helsinki.

A comprehensive set of variables was collected for all included cycles, including general demographic characteristics (female age, male age, infertility duration, body mass index [BMI]), baseline endocrine parameters (basal FSH, AMH), gonadotropin (Gn) stimulation-related indices (Gn stimulation dose, duration of Gn use, total Gn consumption), hormone levels on the day of HCG administration (E2, progesterone [P], LH), oocyte and sperm quality parameters (total oocyte yield, sperm concentration, sperm motility, normal sperm morphology rate, post-processing sperm concentration), and infertility-related etiological factors (primary/secondary infertility, tubal/non-tubal factors). Based on the fertilization outcomes, patient cycles were divided into two groups: cycles with complete fertilization failure and low fertilization (2PN rate < 30%) as the experimental group and cycles with normal fertilization (2PN rate ≥ 30%) as the control group. This grouping is designed to distinctly compare the differences in various parameters under different fertilization conditions, providing a clear framework for subsequent analysis.

### Univariate analysis:

Univariate analysis was performed to compare all collected variables between the fertilization failure group and the normal fertilization group, aiming to screen for potential factors associated with fertilization failure. The analysis focused on exploring the potential correlations between fertilization outcome and variables including female/male age, infertility duration, BMI, baseline endocrine parameters (FSH, AMH), Gn stimulation regimens, hormone levels on the day of HCG administration, oocyte and sperm quality indices, and infertility etiologies. This step was designed to initially identify all variables with potential clinical relevance to fertilization failure from a comprehensive perspective.

### Multivariate logistic regression analysis:

Factors with a statistically significant difference (P<0.05) in the univariate analysis were further included in a multivariate logistic regression model. This analysis was used to adjust for potential confounding effects between variables and to accurately identify the independent risk factors that have a direct impact on fertilization failure in IVF-ET cycles.

### Statistical analysis:

All statistical analyses were performed using the Statistical Package for the Social Sciences (SPSS) version 29.0 software. For continuous variables, the normality of distribution was first tested: independent samples t-test was used for normally distributed data, and non-parametric test was used for non-normally distributed data. For categorical variables, chi-square test or Fisher’s exact probability method was applied for comparison between groups. Factors with P < 0.05 in the univariate analysis were enrolled in the multivariate logistic regression model to calculate the odds ratio (OR) and 95% confidence interval (95% CI). Stepwise regression method was used in the logistic regression analysis to eliminate collinear factors and ensure the reliability of the model results. A two-sided P < 0.05 was considered statistically significant for all analyses.

## RESULTS

A total of 1,818 cycles of IVF–ET treatment were included in this study, consisting of 218 cycles in the experimental group (group with adverse outcomes associated with fertilization failure) and 1,600 cycles in the control group (normal fertilization group). Univariate analysis results showed that the average infertility duration in the experimental group was (4.19±2.87) years, which was significantly longer than that in the control group [(3.61±2.87) years, P = 0.006], suggesting that prolonged infertility duration may be a potential associated factor of fertilization failure. No statistically significant differences were observed in BMI, female age, male age, baseline FSH level, and AMH level between the two groups (all P>0.05). Detailed univariate analysis results for gonadotropin-related indices, hormone levels on the day of HCG administration, oocyte and sperm parameters, and infertility causes are shown in Table-I.

**Table-I:** Univariate analysis and comparison, measurement data = (*x̅*+*s*), count data = n (%).

Item	Fertilization Failure Group (n = 218)	Normal Fertilization Group (n = 1,600)	P
Female Age	33.56±5.14	33.39±5.06	0.649
Male Age	34.55±5.76	34.17±5.31	0.337
Duration of Infertility	4.19±2.87	3.61±2.87	0.006
BMI	24.37±4.37	24.49±4.34	0.697
Baseline FSH	7.16±3.48	6.76±2.97	0.119
AMH	2.93±3.22	3.25±3.09	0.155
% Primary Infertility	120 (55.0%)	646 (40.4%)	<0.001
% Tubal Factors	112 (51.38%)	1137 (71.06%)	<0.001
Gn Stimulation Dose	240.54±67.37	243.87±64.49	0.477
Duration of Gn Use	10.82±2.65	10.97±2.29	0.403
Total Gn	2641.80±1030.98	2704.73±947.82	0.394
E2 on HCG Day	9422.87±8617.26	12310.10±9958.42	<0.001
LH on HCG Day	3.77±4.52	2.91±2.65	0.004
P on HCG Day	3.38±7.79	3.80±8.68	0.254
Total Egg Count	8.80±6.94	10.95±7.60	<0.001
Sperm Concentration	83.76±81.53	93.83±82.23	0.090
Sperm Motility	38.00±18.31	41.79±18.12	0.004
Normal Sperm Morphology Rate	4.79±2.21	5.33±2.41	0.003
Post-treatment Sperm Concentration	9.78±5.38	11.08±5.35	<0.001

Factors with P<0.05 in the univariate analysis were enrolled in the multivariate logistic regression model, and the results (shown in Table-II) identified three independent risk factors for fertilization failure in IVF-ET cycles: primary infertility, non-tubal infertility factors, and LH levels on the day of HCG administration. These three factors were confirmed to have a direct and independent impact on fertilization outcome after adjusting for potential confounding effects of other variables.

**Table-II:** Multivariate Logistic Regression Analysis.

	B	SD	Wald	DF	Significance	Exp(B)	EXP(B) 95% CI	
							Lower Limit	Upper Limit
Primary Infertility (1)	-.377	.177	4.517	1	.034	.686	.485	.971
Duration of Infertility	-.017	.029	.355	1	.551	.983	.929	1.040
Tubal Factors (1)	.720	.176	16.770	1	<.001	2.054	1.456	2.899
LH on HCG Day	-.086	.024	12.583	1	<.001	.917	.875	.962
Total Egg Count	.009	.019	.209	1	.648	1.009	.972	1.047
Sperm Concentration	.001	.001	.626	1	.429	1.001	.999	1.003
PR	.006	.005	1.443	1	.230	1.006	.996	1.016
Normal Sperm Morphology Rate	.075	.044	2.879	1	.090	1.078	.988	1.176
Post-treatment Sperm Concentration	.028	.017	2.710	1	.100	1.029	.995	1.064
E2 on HCG Day	.000	.000	2.209	1	.137	1.000	1.000	1.000
Constant	.751	.374	4.040	1	.044	2.119		

## DISCUSSION

Primary infertility, with its complex, dual-partner etiology involving unrecognized gamete interaction defects and molecular-level reproductive dysfunction,^4^,^5^ was identified as an independent risk factor for IVF-ET fertilization failure in our study, with a 55.0% prevalence in the failure group versus 40.4% in the normal fertilization group (P<0.001). This aligns with Tian T et al.’s large-scale analysis of 106,728 cycles, which confirmed primary infertility as a strong predictor of fertilization disorders.^6^ Conversely, tubal factors emerged as a protective factor (Exp(B)=2.054, 95% CI:1.456-2.899), consistent with Xia Q et al.,^7^ as tubal infertility reflects transport dysfunction rather than impaired sperm-egg fusion abilit,^8^ while non-tubal factors (e.g., unexplained infertility, mild male factor) were verified as an independent risk factor, supporting Li M et al.’s fertilization failure prediction model findings.^9^ A conflicting result from Massarotti C et al.^4^ who found no link between unexplained infertility and fertilization failure likely stems from their inclusion of IVF and ICSI cycles, whereas our study strictly focused on natural sperm-egg fusion, eliminating ICSI’s artificial activation confounding effect.^10^

A novel finding of our study is that primary infertility and non-tubal factors exert independent fertilization effects beyond gamete quality, after adjusting for oocyte count, sperm motility and hormonal parameter.^11^ This complements prior research that only analyzed infertility type as a categorical variable, providing a new clinical lens for identifying high-risk populations beyond traditional gamete quality assessments.^12^

Our study found significantly higher HCG-day LH levels in the fertilization failure group (3.77±4.52 IU/L vs. 2.91±2.65 IU/L, P=0.004), with elevated LH identified as an independent risk factor (Exp(B)=0.917, 95% CI:0.875-0.962). This aligns with Wang Q et al.^13^ and Zhang Q et al.,^14^ who linked elevated LH to low fertilization rates and impaired oocyte quality, likely via premature follicular luteinization that disrupts granulosa-oocyte crosstalk and oocyte fusion ability.^15^,^16^ A conflicting result from Zhang W et al.^17^ who found no LH effect in young women with normal ovarian reserve—reflects population heterogeneity; our mixed ovarian reserve cohort clarifies LH’s pronounced impact in suboptimal reserve patients, a novel addition to existing literature.^18^

Notably, prior research focused on LH’s link to pregnancy outcomes, while our study is the first to confirm its direct, independent association with fertilization failure (adjusting for oocyte count, E2 and sperm parameters).^14^ This fills a critical research gap, linking ovulation-trigger hormonal parameters directly to fertilization and providing a measurable indicator for guiding individualized ovarian stimulation.^19^

The core novelty of this study is the identification of three clinically actionable, independent risk factors (primary infertility, non-tubal factors, elevated HCG-day LH) for conventional IVF fertilization failure, via rigorous multivariate regression that excludes confounding from age, BMI and gamete quality.^12^ Unlike prior mixed IVF/ICSI cohort studies,^6^,^20^ our focus on natural sperm-egg fusion cycles ensures results are directly applicable to clinical conventional IVF practice.^10^ Additionally, we are the first to stratify tubal/non-tubal factors as an independent predictor, moving beyond simple primary/secondary infertility classification for more precise high-risk stratificatio.^9^

### Limitations:

This single-center retrospective study has specific limitations: first, selection bias from restricting to first-cycle natural fusion patients limits generalizability to repeat cycles or mild stimulation regimens; second, unanalyzed molecular/biological factors (sperm DFI, oocyte morphology, endometrial markers) may limit model comprehensiveness; third, no optimal LH cut-off value was determined, hindering immediate clinical intervention guidance; fourth, only fertilization outcomes were assessed, with no analysis of subsequent implantation or live birth rates; fifth, psychological factors (anxiety/depression) were not accounted for, despite their potential interaction with LH levels.

## CONCLUSIONS

Elevated LH levels on the day of HCG administration and primary infertility with non-tubal etiologies are key independent risk factors for fertilization failure in IVF-ET cycles. Close clinical monitoring and targeted early interventions for these high-risk factors are vital to mitigate fertilization failure and optimize IVF-ET treatment outcomes.

### Recommendations:

Future large-scale, multi-center prospective studies are needed to validate these risk factors, define LH cut-offs and evaluate targeted interventions (e.g., GnRH antagonist adjustment, half-ICSI for high-risk cases) to reduce fertilization failure rates.

### Authors’ Contributions:

**JL:** Study design, literature search and manuscript writing.

**YQ, QS** and **CL:** Data collection, data analysis and interpretation. Critical Review.

**RL** and **HS:** Manuscript revision and validation, Critical Analysis.

All authors have read, approved the final manuscript and are responsible for the integrity of the study.
